# A randomised controlled trial of nurse-managed trial conclusion following early phase cancer trial participation

**DOI:** 10.1038/sj.bjc.6602675

**Published:** 2005-06-28

**Authors:** K Cox, E Wilson, A Arthur, R Elkan, S Armstrong

**Affiliations:** 1 School of Nursing, University of Nottingham, Faculty of Medicine and Health Sciences, University of Nottingham, Nottingham NG7 2UH, UK; 2 Trent Institute for Health Services Research, University of Nottingham, Faculty of Medicine and Health Sciences, University of Nottingham, Nottingham NG7 2UH, UK

**Keywords:** clinical trials, nurse-managed care, randomised controlled trial, trial conclusion

## Abstract

The effect of a nurse-managed intervention, for early phase cancer trial participants at trial conclusion, on psychosocial outcomes was evaluated at two cancer centres in the Midlands, England using a randomised controlled trial. It involved 117 patients who were participating in an early phase cancer clinical trial. It was a nurse-managed trial exit, which included a trial exit interview, trial feedback information leaflet and telephone follow-up compared with standard care at trial conclusion. Psychological distress at 1 week and 4–6 weeks post-trial conclusion, patient's knowledge and understanding and patient's satisfaction were assessed. The results showed there was no significant difference between the two groups regarding scores for anxiety and depression at time one and time two. There is some suggestion that the intervention reduced anxiety from trial conclusion to follow-up (*P*=0.27). Patients in both groups felt they had contributed to cancer research through trial participation. However, intervention patients were more likely to feel that they knew how the trial was going (*P*<0.001), knew how other people in the trial were doing (*P*=0.001), had all the feedback they needed about the trial they took part in (*P*<0.01) and knew how they would be followed up (*P*=0.02). Patient satisfaction with the intervention was high (median score=4.5 where 5 is greatest satisfaction). In conclusion, nurse-managed trial conclusion led to positive outcomes for patients who had recently completed a clinical trial.

Participation in clinical trials of new anticancer drugs has become an increasingly common treatment experience of individuals with cancer. This is due, in part, to an increasing societal demand for new treatments and the need to test these treatments in a systematic way (DOH, 1992). However, until recently there has been relatively little attention paid to the impact of the experience of clinical trials on those who participate in them (Mackillop and Johnston, 1986; Gotay, 1991; Kodish *et al*, 1992). There is evidence from qualitative research that trial conclusion is the most difficult time for the subjects of clinical research, who often feel abandoned, want feedback about the trial they took part in and have unmet information and psychosocial needs (Cox, 2000).

The management of trial conclusion is a neglected area. Staff resource in terms of support, time and information is directed at the stage of trial recruitment and trial participation. However, trials staff have an ethical responsibility to ensure appropriate support for those who have been research participants (Harth and Thong, 1995).

One of the ways individuals cope with cancer is that they seek more information (Weisman, 1979). If information is given effectively, anxiety and side effects of treatment can be reduced, enhancing an individuals ability to cope with their illness (Ridgeway and Matthews, 1982; Slevin *et al*, 1996). Recognising and meeting the very specific information and support requirements at trial conclusion may be one way that on-going psychosocial support can be provided to trial patients and enable them and their families to cope better with trial conclusion. The study presented in this paper sought to provide an alternative model of trial conclusion management, which responded to the issues identified above and establish if this improved the trial conclusion experience for patients. In this study, trial conclusion is defined as the point in time when a patient has completed the planned course of treatment or they were withdrawn due to unacceptable toxicity or lack of response.

## METHODS

The study was conducted in two cancer centres. All patients who were currently undergoing participation in all the phase I or II anticancer drug trials underway in the two centres were invited to participate via a letter and information sheet given to them by their trials nurse. Those who agreed provided signed consent. It should be noted that patients offered phase I and II studies are often at the end of their disease trajectory and as such the patients in this study had a limited life expectancy. Ethical approval and NHS Trust approval was granted at each study site.

### Randomisation

A computer-generated list of random codes using block randomisation was generated by SA and held by a research secretary. Participants to our study were randomly allocated following recruitment to the study. Each participant had an equal chance of being allocated to the intervention group (nurse-managed follow-up) or the control group (standard care).

### Intervention

The intervention was developed in the light of findings from our earlier work (Cox, 2000), discussions with consultant oncologists, trials nurses and patients and families who were asked if they would comment on ideas and information sheets. This preliminary work and the findings from the earlier study indicated that the intervention would consist of three elements (see Box 1). In addition to standard care, the trial exit interview and feedback leaflet were delivered in the week following trial conclusion and the telephone call was undertaken 2 weeks after trial conclusion.

The trial nurses who were to be involved in delivering the intervention all underwent a short training session to ensure their understanding of the process of undertaking the trial exit interview, how to complete the trial feedback sheets and how to conduct the telephone interviews. These training sessions were led by KC and EW and were designed to identify any problems with the intervention, make sure the documentation was appropriate and also to ensure that there was a consistency in its implementation.

### Standard care

For patients allocated to standard care, information, support and follow-up was offered at the end of the drug trial by their trials doctor and consisted of a consultation that covered details of reasons for trial conclusion and a monthly follow-up appointment back at the cancer centre.

### Outcome measures

Outcome measures were psychological distress at 1 week and 4–6 weeks post-trial conclusion, patient's knowledge and understanding and patient's satisfaction. These were recorded either at the patient's home or the cancer centre according to patient preference approximately 7–10 days following trial conclusion and after the intervention for the intervention group (time one) and approximately 6 weeks following trial conclusion (time two).

#### Time one

An evaluation questionnaire was specifically designed for the study. The questionnaire collected data on patients’ experience of trial conclusion and follow-up, their information requirements, knowledge and understanding of the trial outcome and their follow-up care, satisfaction with trial involvement and their care during the trial and adjustment to no longer being in the trial. Questions ranged from fixed choice, rating scales and likert scales to open comments. Patients either self-completed or completed the form with the researcher according to their personal preference.

Psychological distress was measured using the Hospital Anxiety and Depression Scale (HADs) (Zigmond and Snaith, 1983) generating individual scores for anxiety and depression, both ranging from 0 to 21 with higher scores indicating a greater degree of anxiety or depression. Psychological and physical distress was also measured using the Rotterdam Symptom Checklist (Pruyn *et al*, 1980; de Haes *et al*, 1990). Psychological and physical distress scores were standardised so they represented a percentage of the maximum score with higher scores representing greater distress. Hopwood *et al* (1991) note that the HADs and the RSCL may screen out different individuals with affective disorders. With these factors in mind it was appropriate to use both the RSCL and the HADs in this study in order to assess psychological distress at trial conclusion. To assess satisfaction with the intervention patients in the intervention arm of the study also completed the Medical Interview Satisfaction Scale (MISS) (Wolf *et al*, 1978). The tool was designed to measure satisfaction with medical consultations/interviews. While this tool is acknowledged as being a relatively crude measure, as responses on these kinds of scales tend to be skewed towards the satisfied (Ware and Hays, 1988), it was felt that it would provide some indication of patient's satisfaction with the trial exit interview at data point one which could then be elaborated on in subsequent interviews at data point two. The reliability and validity of the tool has not been extensively tested but Kinnersley *et al* (1996) noted in a comparison of methods to measure satisfaction with consultations in primary care that levels of reliability for the overall scale and subscales was fair to good for the MISS. The tool was adapted for use in this study to refer to nurses rather than doctors and has 26 items that measure satisfaction with the affective, behavioural and cognitive aspects of medical encounters. The adaptation primarily involved substituting the word doctor with nurse and substituting reference to being ill to being in a trial.

#### Time two

Patients completed repeat HADs and RSCL questionnaires and took part in an in-depth interview (not reported here) examining patients’ experience of trial conclusion and follow-up, their knowledge and understanding of the trial outcome and their follow-up care, satisfaction with trial involvement and their care during the trial and adjustment to no longer being in the trial.

### Sample size

Based on previous work (Cox, 2000) at the time of trial conclusion, 38% of patients had mild to severe anxiety as assessed by the HAD scale. It was estimated that through the intervention, this would be decreased by just over half to 15% at time one (taking into account reported prevalence rates for anxiety and depression in populations of cancer patients of around 17% (Derogatis *et al*, 1983)). The required number of patients in each arm of the study was 57 (*α*=0.05 (two sided) with 80% power, Machin *et al*, 1997).

### Statistical methods

Differences between scores on information received, information requirements, knowledge and understanding of the trial outcome, anxiety and depression, psychological and physical distress were compared between groups using the Mann–Whitney *U* test because the distribution of the scores was skewed.

## RESULTS

**Figure 1:**
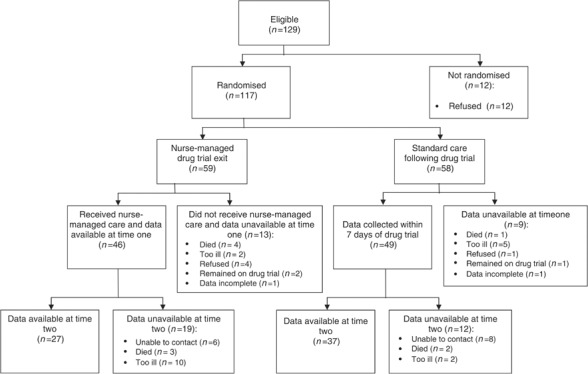
Flow of participants through trial.

Figure 1 reports the flow of participants through the study. Between 1 January 2001 and 1 February 2004, 129 patients were approached about participating in the study. A total of 12 chose not to be involved and the remaining 117 (91%) agreed to be part of the study providing signed written consent. These were randomly allocated to either the nurse-led follow-up group (*n*=59) or standard care (*n*=58). Measures at time one were collected on 46 out of 59 of the intervention group and 49 out of 58 of the control group. The main reasons for drop out were refusal, too ill to participate and death.

Demographic and other characteristics for the two study groups at drug trial conclusion are reported in Table 1. More men were included in the study than women, the average age was just under 60 years and the majority was married. Patients were likely to have one of the common solid tumours. Patients were more likely to have been withdrawn from the trial in which they were participating. There was an equal distribution of patients in both groups across both research sites. There were no obvious differences between the two groups.

### Anxiety and psychological distress

Table 2 presents the HADS median scores for anxiety and depression and the RSCL median scores for psychological and physical distress at the two data collection points for both the intervention and the control groups. The median scores for both anxiety and depression for these patients over the course of trial participation were within the normal range for both the HADs (0–7) (Zigmond and Snaith, 1983). The average scores for psychological distress for these patients were comparable with those published by de Haes *et al* (1990) for patients receiving chemotherapy. There was no significant difference between the two groups regarding scores for anxiety and depression, psychological distress or physical distress at trial conclusion or follow-up. In the intervention group there was a greater reduction in the anxiety scores from baseline to follow-up (−1.2) compared to the control group (−0.6), although this was not statistically significant (*P*=0.27).

### Knowledge and understanding of the trial outcome and follow-up

Table 3 presents respondents views on information needs and contribution made to cancer research. There were significant differences between those in the intervention group when compared to those in the control group in relation to the statements; I feel I know how the trial is going (*P*<0.001), I feel I know how other people in the trial are doing (*P*=0.001), I feel I have had all the feedback that I need about the trial I took part in (*P*<0.01) and I feel I know how I will be followed up now I am no longer in the trial (*P*=0.02) with those in the intervention group indicating significantly higher levels of agreement with the statements. Interestingly, both groups felt that they had contributed to cancer research by taking part in the trial with no difference between the groups (*P*=0.11).

### Satisfaction with the intervention

A total of 44 respondents in the intervention group (from a possible 47) completed the MISS. Satisfaction with all elements (cognitive, emotional and behavioural) of the trial exit interview was good. Overall patient satisfaction with the intervention was high (median score=4.5 where 5 is greatest satisfaction).

## DISCUSSION

An individuals desire for ‘feedback’ and the provision of information about how the trial is going can be seen in the context of the process of adaptation within the context of life threatening situations (Turnquist *et al*, 1988), a kind of ‘search for meaning’. Research in this area suggests that an individual's ability to search for and find a meaning in their illness and treatment may have a significant impact on psychosocial well-being and adjustment to the impact of cancer on their lives (Lewis, 1989; Luker *et al*, 1996). Providing feedback and information about the trial, an individual has participated in may be one way that on-going psychosocial support for patients can be offered and the contribution made through their trial participation acknowledged. This requirement is even more important in this particular group of patients who are at the end of their disease trajectory, have a limited life expectancy, have complex needs and who are participating in a drug trial that is unlikely to have any therapeutic benefit (Estey *et al*, 1986; Marsoni *et al*, 1987; Decoster *et al*, 1990). With this underpinning rationale we designed an intervention to meet these needs. The findings show that nurse-managed trial conclusion meets patients’ information needs at trial conclusion. The intervention was acceptable to patients and this study supports previous work in relation to the acceptability of nurse-managed follow-up and satisfaction with this kind of care delivery (Moore *et al*, 2002). It would appear that, while there was no significant difference between the intervention and control group in terms of anxiety and depression levels at base line and follow-up, anxiety was reducing over time for those individuals in the intervention arm of the study but this requires further testing. Overall, the intervention was effective in providing patients with information and feedback about the trial that had been identified as a specific need at trial conclusion in our earlier work.

One key limitation of this study is the lack of power as a result of the high attrition between the two time points. This was perhaps inevitable due the high mortality rate associated with this group of patients. It may also be a reflection of an overestimation of the effect of the intervention in our initial sample size calculations. A further limitation relates to the mode of completion of the questionnaires. Patients completing their questionnaires with the help of a researcher may provide different responses to those who complete alone.

## CONCLUSIONS

Providing structured support and information at the end of a trial ensures information needs are met and is acceptable to patients. The trial conclusion strategy outlined here would therefore appear to be a simple and effective way to provide on-going psychosocial support to patients once the trial has completed. It can bridge the gap between the end of the trial and the next follow-up appointment and make a difference to the trial conclusion experience of vulnerable patients.

## Figures and Tables

**Table 1:** Demographic factors, tumour and clinical trial type by study group

	**Intervention group (*n*=59)**	**Control group (*n*=58)**
*Gender*		
Male : female	1.1 : 1	1.4 : 1
		
*Age in years*		
Mean (s.d.)	59.8 (11.6)	57.4 (10.8)
		
*Marital status n* (%)		
Living with spouse/partner	53 (89.8)	45 (81.8)
Divorced	3 (5.1)	3 (5.5)
Widowed	1 (1.7)	4 (7.3)
Single	2 (3.4)	3 (5.5)
Missing		3
		
*Tumour type n* (%)		
Breast	7 (12.1%)	5 (8.6%)
Lung	8 (13.8%)	7 (12.1%)
Upper GI	7 (12.1%)	10 (17.2%)
Ovary	7 (12.1%)	5 (8.6%)
Colorectal	11 (19.0%)	11 (19.0%)
Other	18 (31.0%)	20 (34.5%)
		
*Drug Trial centre n* (%)		
Centre 1	52 (88.1%)	51 (87.9%)
Centre 2	7 (11.9%)	7 (12.1)
		
*Completed drug trial n* (%)		
Yes	17 (28.8%)	20 (34.5%)
No	42 (71.2%)	38 (65.5%)
		
*Days spent on drug trial*		
Mean (s.d.)	96.8 (68.2)	97.8 (54.0)

**Table 2:** Anxiety, Depression Physical Distress and Psychological Distress scores within 7 days of trial exit and after 4–6 weeks

		**Intervention mean (s.d.)**	**Control mean (s.d.)**	***P*-value^a^**
HADS Anxiety Scores	Within 7 days	4.7 (4.2) (*n*=46)	4.8 (3.9) (*n*=49)	
	4–6 weeks	3.6 (3.8) (*n*=27)	4.4 (3.7) (*n*=37)	
	Change over time	−1.2 (3.6) (*n*=27)	−0.6 (2.7) (*n*=37)	0.27
				
HADS Depression Scores	Within 7 days	5.2 (4.3) (*n*=46)	4.9 (3.3) (*n*=49)	
	4–6 weeks	5.3 (5.0) (*n*=27)	4.7 (3.8) (*n*=37)	
	Change over time	0.2 (2.7) (*n*=27)	−0.2 (3.3) (*n*=27)	0.51
				
RSCL Physical Distress Scores	Within 7 days	23.8 (13.8) (*n*=46)	20.4 (10.5) (*n*=49)	
	4–6 weeks	20.4 (14.7) (*n*=27)	18.9 (13.3) (*n*=37)	
	Change over time	−2.6 (13.8) (*n*=27)	−2.2 (14.0) (*n*=37)	0.73
				
RSCL Psychological Distress Scores	Within 7 days	21.8 (24.0) (*n*=46)	23.4 (18.9) (*n*=49)	
	4–6 weeks	18.9 (20.3) (*n*=27)	23.0 (20.4) (*n*=37)	
	Change over time	−2.3 (20.4) (*n*=27)	−2.3 (18.4) (*n*=37)	0.49

a Between groups, Mann–Whitney *U* test.

**Table 3:** Views on information needs and perception of contribution by study group (scores out of five, higher scores indicate greater agreement)

	**Intervention group (*n*=46) median (IQR)**	**Control group (*n*=49) median (IQR)**	***P*-value^a^**
I have contributed to cancer research	5 (5,5)	5 (5,5)	0.11
I know how the trial is currently going	5 (3.75,5)	1 (1,4)	<0.0005
I know how other people on the trial are doing	4 (1,5)	1 (1,4)	0.001
I have had all the feedback that I need about the trial I took part in	5 (4,5)	4 (1,5)	0.009
I know how I will be followed up now I am no longer in the trial	5 (5,5)	5 (1,5)	0.022

a Mann–Whitney *U* test.

**Box 1:** Key components of nurse-managed trial exit intervention

*Drug trial exit interview*
•Debriefing of decision around completion/withdrawal
•Explanation of further follow-up support

*Information leaflet*
•A thank you for participating in the trial
•Latest information about the drug being tested
•News about other participants
•Details about the participant's contribution to cancer research
•Available support after drug trial participation
•Details on further follow-up

*Telephone follow-up at 2 weeks postdrug trial exit*
•Enquiry as to general health
•Identification of unmet information needs
•Emotional support if required
